# Deep breathing exercises in easing educational stress among Indian high school students

**DOI:** 10.6026/973206300200156

**Published:** 2024-02-29

**Authors:** B Mahalakshmi, Katara Mittalben Ghemabhai, Amita Shilpa Gottlieb, N Sivasubramanian, Padmavathi Parthasarathy

**Affiliations:** 1 Department of Paediatric Nursing, Nootan College of Nursing, Sankalchand Patel University, Visnagar, Gujarat - 384315, India; 2 Department of obstetric and gynaecological Nursing, Graphic Era College of Nursing, Graphic Era Deemed to be University, Dehradun, Uttarakhand - 248002, India; 3 Department of Psychiatric Nursing, Nootan College of Nursing, Sankalchand Patel University, Visnagar, Gujarat - 384315; India; 4 Department of Biochemistry, Nootan Medical College & Research Centre, Sankalchand Patel University, Visnagar, Gujarat, India

**Keywords:** Educational stress, higher secondary students, deep breathing exercises

## Abstract

In this educational landscape, the pervasive issue of stress among higher secondary students has emerged as a serious matter. Acknowledging the challenges posed by educational stress, this research explores holistic and accessible interventions, with a focus on the promising avenue of deep breathing exercises known for their effectiveness in promoting relaxation and reducing stress. The study employs a quasi-experimental design, comparing an experimental group engaged in daily deep breathing exercises with a control group following a regular routine. Sixty higher secondary students in Visnagar participate through purposive sampling, adhering to specific inclusion criteria. The intervention includes a pre-tested questionnaire to assess stress levels, the implementation of daily deep breathing exercises in the experimental group, and a post-intervention stress level reassessment in both groups. The findings reveal a notable reduction in stress levels post-intervention, particularly in the experimental group practicing deep breathing exercises. Statistically significant reductions in mean stress scores underscore the effectiveness of this intervention, with the experimental group demonstrating a significantly lower mean stress score compared to the control group. This study contributes vital insights into stress management strategies for higher secondary students, highlighting the efficacy of incorporating deep breathing exercises into their routine. The observed reductions in stress levels emphasize the potential benefits of practical stress reduction techniques within the educational milieu.

## Background:

In the educational settings, the prevalence of academic stress among higher secondary students has become more common and main core of concern. [1] The demanding academic environment, coupled with societal expectations, places a significant burden on students, potentially hindering their overall well-being and academic performance. [2] Recognizing the need for effective stress management strategies, this study investigates the impact of deep breathing exercises as a potential intervention to alleviate educational stress. [3] The educational journey of higher secondary students is often marked by rigorous academic demands, competitive atmospheres, and the need to excel across diverse domains. [4] In the pursuit of academic excellence, students frequently encounter stressors that can adversely affect their mental and emotional health. Educational stress has been associated with various negative outcomes, including reduced cognitive functioning, compromised mental health, and impaired academic performance. [5] Addressing the challenges posed by educational stress necessitates the exploration of holistic and accessible interventions. Deep breathing exercises, renowned for their effectiveness in promoting relaxation and reducing stress, emerge as a promising avenue for intervention. [6] Therefore, it is of interest to assess the educational stress levels among higher secondary students and evaluate the effectiveness of incorporating deep breathing exercises into their routine.

## Methodology:

## Design:

Quasi-experimental design comparing an experimental group (deep breathing exercises) with a control group

## Participants:

60 higher secondary students from Nootan Sarv Vidhyalaya in Visnagar

## Sampling:

Purposive sampling with specific inclusion criteria

## Intervention:

Experimental group engages in daily deep breathing exercises; control group follows regular routine.

## Inclusion and Exclusion Criteria:

Participants meeting the inclusion criteria, i.e., higher secondary students from Nootan Sarv Vidhyalaya in Visnagar, were considered for the study. Inclusion criteria encompassed students willing to participate in the deep breathing exercise intervention. Exclusion criteria involved individuals with pre-existing respiratory conditions or those unwilling to engage consistently in the prescribed activities. This ensured a homogeneous sample for a focused investigation into the effectiveness of deep breathing exercises on educational stress.

## Data Collection:

[1] Assessment: Pre-tested questionnaire measuring educational stress.

[2] Implementation: Deep breathing exercises in the experimental group.

[3] Post-Intervention: Re-assessment of stress levels in both groups.

## Analysis:

Descriptive and inferential statistics, including t-tests.

## Ethics:

Adherence to ethical principles, with approval from the ethical review board.

## Results:

Table 1 provides an insightful breakdown of the demographic composition of the study participants from NootanSarvVidhyalaya in Visnagar. Among the higher secondary students, the age distribution reveals that the majority (56.7%) fall within the 16-17 age group, with a relatively even gender distribution. Religiously, all participants identified as Hindu. Socio-economic status predominantly leans towards the medium category, and the majority of students reported studying for 3-4 hours daily due to parental force. Interestingly, more than half of the participants in both groups had previous experiences of educational stress. In the experimental group, 26.7% reported low stress, 50.0% mild stress, and 70.0% moderate stress post-intervention. Similarly, the control group experienced reductions, with 26.7% in low stress, 50.0% in mild stress, and 63.3% in moderate stress. Statistically significant reductions in mean stress scores were observed. The control group exhibited a significant decrease from 120.60 to 107.10 (p < 0.05), and the experimental group showed a highly significant drop from 123.10 to 74.97 (p < 0.05). Importantly, the experimental group, engaged in deep breathing exercises, achieved a notably lower mean stress score (48.13) compared to the control group (13.50), indicating a more pronounced effect of the intervention. This significant difference (p < 0.05) underscores the effectiveness of deep breathing exercises in reducing educational stress. Table 2 examined the association between occurrences of academic stress and demographic variables in both the experimental and control groups. In the control group, a significant link was found between sex and stress (χ² = 7.48, df = 2, p < 0.05), suggesting gender-specific aspects of stress. Socio-economic status also showed significance (χ² = 1.24, df = 2, p < 0.05) in the control group, indicating its influence on academic stress. However, these associations were not present in the experimental group, hinting at the potential mitigating effect of deep breathing exercises on gender and socio-economic influences on academic stress. The lack of significance in the experimental group for sex (χ² = 4.19, df = 2, p > 0.05) and socio-economic status (χ² = 0.172, df = 2, p > 0.05) suggests that deep breathing exercises may offer equitable stress relief, regardless of gender or economic background.

## Discussion:

Our study, focused on evaluating the effectiveness of deep breathing exercises on educational stress among higher secondary students at NootanSarvVidhyalaya in Visnagar, unveils crucial insights into stress management strategies. The objectives encompassed assessing educational stress prevalence, gauging the impact of deep breathing exercises, and drawing comparisons between an experimental group exposed to the exercises and a control group. Our findings resonate with the work of Sandal *et al.*, 2017, revealing a 47% prevalence of educational stress among higher secondary students. This consistency underscores the persistent nature of educational stress and aligns with broader literature in the field. [7,8, [Bibr R09]] Implementation of deep breathing exercises demonstrated positive outcomes, showcasing a significant decrease in stress levels. These results parallel those of Valentina *et al.* reinforcing the potential efficacy of deep breathing exercises in alleviating educational stress among higher secondary students.[10]

**Figure 1:**
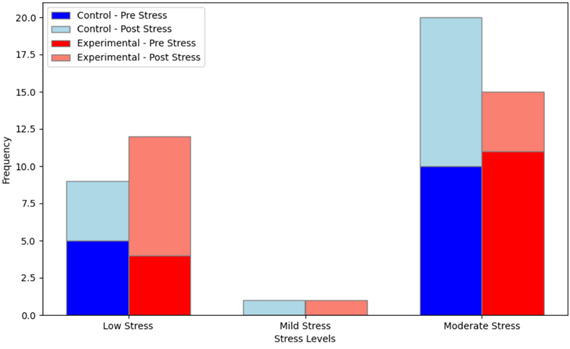
Pre/post stress scores in control and experimental groups

Upon comparing the experimental and control groups, notable differences emerged, indicating that deep breathing exercises have a discernible impact on reducing academic stress compared to traditional methods (Figure 1). This outcome supports and extends the findings of Naik *et al.*, 2018 and hopper *et al.*, 2019] [11,12], who reported similar results in their study. These variations may stem from differences in sample size, methodology, or demographic characteristics, highlighting the need for nuanced interpretations and potential avenues for future research. Regarding age, no significant association with academic stress in either group, echoing findings from studies such as Siti *et al.* 2020 and Mona *et al.* 2020, which similarly reported a lack of correlation between age and stress levels [13]. This suggests that the experience of academic stress transcends age groups, emphasizing the necessity of interventions tailored to the individual needs of students, irrespective of their age. Socio-economic status emerged as a notable factor, showing a significant association with academic stress in the control group but not in the experimental group. This aligns with findings from Wu *et al.* 2022, suggesting that socio-economic factors can influence stress levels [14]. The lack of this association in the experimental group may signify the effectiveness of interventions specifically designed to mitigate socio-economic influences on academic stress. In examining the hours of study, no significant association was found in the experimental group, in contrast to studies like Yuwei *et al.* 2022, which reported such associations. This discrepancy may stem from variations in study methodologies or differences in the demographics of the student populations under investigation. [15]

## Conclusion:

Daily deep breathing exercises proved highly effective in significantly reducing educational stress among higher secondary students, as evidenced by a lower mean stress score in the experimental group. These findings highlight the practical utility of incorporating accessible stress reduction techniques in educational settings. The observed benefits underscore the potential impact of such interventions on the overall well-being of students. Implementing similar strategies could contribute to more comprehensive stress management approaches in educational environments.

## Figures and Tables

**Table 1:** distribution of sample as per demographic variables

**Subcategory**	**Experimental (Frequency, Percentage)**	**Control (Frequency, Percentage)**
**Age**		
15 - 16	5 (16.7%)	7 (23.3%)
16 - 17	17 (56.7%)	21 (70.0%)
17 - 18	8 (26.7%)	2 (6.7%)
**Sex**		
Male	15 (50.0%)	12 (40.0%)
Female	15 (50.0%)	18 (60.0%)
Religion		
Hindu	30 (100.0%)	30 (100.0%)
Socio-Economic Status		
High	3 (10.0%)	2 (6.7%)
Medium	27 (90.0%)	28 (93.3%)
**Hours of Study**		
3 - 4 Hours	26 (86.7%)	28 (93.3%)
4 - 6 Hours	4 (13.3%)	1 (3.3%)
**Study Due to Parental Force**		
Yes	14 (46.7%)	18 (60.0%)
No	16 (53.3%)	12 (40.0%)
**Previous Exp. of Edu. Stress**		
Yes	17 (56.7%)	18 (60.0%)
No	13 (43.3%)	12 (40.0%)

**Table 2:** Association between Occurrences of Academic Stress and Demographic Variables

**Demographic Variable**	**Control Chi-Square Value**	**Control DF**	**Experimental Chi-Square Value**	**Experimental DF**
Age in Year	1.37 (Non-significant)	4	3.392 (Non-significant)	4
Sex	7.48 (significant)	2	4.19 (Non-significant)	2
Religion	-	-	-	-
Socio-Economic Status	1.24 (Non-significant)	2	0.172 (Non-significant)	2
Hours of Study	1.24 (Non-significant)	4	1.36 (Non-significant)	4
Study Due to Parental Force	3.84 (Non-significant)	2	9.265 (significant)	2
Previous Exp. of Edu. Stress	0.111 (Non-significant)	2	2.56 (Non-significant)	2
At 0.05 level of significant
